# Design and development of novel non-peptide agonists at NPR-C

**DOI:** 10.1186/1471-2210-11-S1-P54

**Published:** 2011-08-01

**Authors:** Filipa Mota Quinteiro, Paul Gane, Anne-Sophie Rebstock, Roberta Worthington, Michela Simone, Snezana Djordevic, Adrian Hobbs, David Selwood

**Affiliations:** 1 Wolfson Institute for Biomedical Research, University College London, London, WC1E 6BT, UK; 2 Institute for Structural and Molecular Biology, University College London, London WC1E 6BT, UK; 3 Neuroscience, Physiology & Pharmacology, University College London, London, WC1E 6BT, UK

## Background

Endothelium-derived C-type natriuretic peptide (CNP) possesses cytoprotective and anti-atherogenic functions that regulate vascular tone and smooth-muscle relaxation and might be key in protecting against ischaemia-reperfusion injury [1]. The finding that many of the vasoprotective effects of CNP are mediated by the natriuretic peptide receptor type-C (NPR-C) suggests that this receptor might represent a novel therapeutic target for the treatment of cardiovascular diseases. Thus, we have designed and developed small molecule drug-like mimetics of CNP agonists at NPRC.

## Methods and results

We employ a multi-disciplinary approach that comprises molecular modelling, chemical synthesis and biological and functional assays. The crystal structure of NPR-C was used as the starting point for the design of peptidic and subsequently non-peptidic ligands to the receptor [2]. We have determined which fragments of CNP are crucial for binding to NPR-C and modified the NPR-C antagonist AP-811 using pharmacophore searches to replace the peptide component, which led to the design of a library of compounds that were subsequently synthesised, tested and optimised [3].

## Conclusion

Novel and selective non-peptide NPR-C agonists have been identified that relax rat isolated mesenteric arteries in vitro. We foresee that such molecules will facilitate the development of potential therapeutic agents for cardiovascular diseases.
